# Predicting complications in pediatric Crohn's disease patients followed in CEDATA-GPGE registry

**DOI:** 10.3389/fped.2023.1043067

**Published:** 2023-02-15

**Authors:** Juliane Klamt, Jan de Laffolie, Elisa Wirthgen, Sebastian Stricker, Jan Däbritz, the CEDATA-GPGE study group

**Affiliations:** ^1^ Rostock Medical School, University of Rostock, Rostock, Germany; ^2^ Department of Pediatrics, Justus Liebig University Giessen, Giessen, Germany; ^3^ Department of General Pediatrics and Neonatology, Rostock University Medical Center, Rostock, Germany; ^4^ Department of Pediatrics, Greifswald University Medical Center, Greifswald, Germany

**Keywords:** prediction, outcome, complication, surgery, disease behavior, growth, hospitalization, perianal disease

## Abstract

**Background:**

Complications of Crohn's disease (CD) often impair patients' quality of life. It is necessary to predict and prevent these complications (surgery, stricturing [B2]/penetrating [B3] disease behavior, perianal disease, growth retardation and hospitalization). Our study investigated previously suggested and additional predictors by analyzing data of the CEDATA-GPGE registry.

**Methods:**

Pediatric patients (< 18 years) diagnosed with CD with follow up data in the registry were included in the study. Potential risk factors for the selected complications were evaluated by performing Kaplan-Meier survival curves and cox regression models.

**Results:**

For the complication surgery, the potential risk factors older age, B3 disease, severe perianal disease and initial therapy with corticosteroids at the time of diagnosis were identified. Older age, initial therapy with corticosteroids, low weight-for-age, anemia and emesis predict B2 disease. Low weight-for-age and severe perianal disease were risk factors for B3 disease. Low weight-for-age, growth retardation, older age, nutritional therapy, and extraintestinal manifestations (EIM) of the skin were identified as risk factors for growth retardation during the disease course. High disease activity and treatment with biologicals were predictors for hospitalization. As risk factors for perianal disease, the factors male sex, corticosteroids, B3 disease, a positive family history and EIM of liver and skin were identified.

**Conclusion:**

We confirmed previously suggested predictors of CD course and identified new ones in one of the largest registries of pediatric CD patients. This may help to better stratify patients’ according to their individual risk profile and choose appropriate treatment strategies.

## Introduction

Pediatric-onset CD is a multisystemic, autoinflammatory disease with rising incidence (up to 21.3 per 100,000 children under the age of 18 years) in western countries (1). Abdominal pain, diarrhea, hematochezia and failure to thrive are the common unspecific initial symptoms in pediatric CD (2). During the disease course different complications such as the need for surgery, a stricturing (B2) or penetrating (B3) disease behavior, perianal disease, growth retardation and hospitalization can occur (3–5). These complications of CD often impair patients' quality of life. Two extensive systematic reviews including meta-analysis, published in 2021 (6, 7) made consensus statements on possible predictors of poor outcome in pediatric CD and ulcerative colitis. However, Ricciuto et al. also mentioned that more longitudinal studies are needed to further characterize potential predictors of complications in pediatric CD (6). The aim of our study was to investigate whether the identified predictors of Ricciuto et al. can be confirmed in the CEDATA-GPGE registry. In addition, we analyzed selected potential predictors in more detail, e.g., extraintestinal manifestations (EIM). Furthermore, new combinations of potential predictors and complications were tested to find unknown risk factors or protective factors. The complications we analyzed, were related to the most important outcomes to predict which were defined by Ricciuto et al. [disease behavior (B2/B3), need for surgery, perianal disease, linear growth impairment, malnutrition, pubertal delay, decreased bone mineral density and hospitalization] (6).

## Patients and methods

### Patient population

This retrospective study analyzed prospectively collected data from the CEDATA-GPGE registry, a large registry of children and adolescents from Germany and Austria with inflammatory bowel disease (IBD). The registry exists since 2004 and includes 5,967 (June 2021) IBD patients from 130 hospitals or practices. Between 2014 and 2021 an average of 266 new patients were included each year. The registry provides detailed information about the disease activity, diagnosis, symptoms, laboratory test results, therapies, growth parameters, age, sex, disease behavior, and EIM. The data of the registry is collected through an online standardized case report form that is completed by the treating pediatric gastroenterologist at the time of diagnosis and follow-up (2).

### Inclusion criteria

The study included CD patients from CEDATA-GPGE registry under the age of 18. CD was diagnosed based on the Porto criteria that include clinical signs, symptoms, endoscopy, histology and radiology (8). Patients who did not have a registered follow-up visit or were followed-up for less than 28 days were excluded. Furthermore, patients had to be registered no later than 90 days after diagnosis.

### Disease complications

The main aim of the study was to analyze possible risk factors for seven frequent complications that might occur during the course of CD. Those complications are stricturing disease (B2 behavior), penetrating disease (B3 behavior), the necessity of surgery, growth retardation (body weight at least two standard deviations below the mean), hospitalization (necessity of stay in hospital, no inclusion of day-care in hospital), perianal disease [perianal fistula, perianal abscess, anal eczema, perianal lesion(s)] and bone disease.

### Predictors of disease course

The following characteristics at diagnosis were verified as possible risk factors: body mass index (BMI, at least two standard deviations below the mean), low weight-for-age (body weight at least two standard deviations below the mean), disease location L4 (upper gastrointestinal tract) according to Paris Classification (9), age, sex, disease behavior (B2, B3), perianal disease (5 stages), specific EIM, a corticosteroid therapy, a therapy with biologics, exclusive enteral nutrition (EEN), family history and specific symptoms. The following EIM were investigated: eye-, joint-, liver-, pancreas- and skin manifestations. The specific symptoms included abdominal pain, activity limitations, anemia, hematochezia, diarrhea, fever, lip-/mouth involvement, loss of appetite, emesis, and weight loss. Instead of a clinical disease activity index, the registry included the variable “physician overall assessment”, which was used to provide a subjective expert assessment of disease activity (four categories: remission, low, moderate and severe activity). The possible predictor growth retardation was defined as at least two standard deviations below the mean. Furthermore, growth retardation was also analyzed as a subjective evaluation provided by the pediatric gastroenterologist in the registry's case report form. Age, disease activity and perianal disease were divided into categories. All other possible predictors were binary variables. Detailed definitions of predictors for complications in pediatric CD patients are provided in supplementary Table 1.

### Statistical methods

SAS version 9.4 was used to transfer the necessary registry data into Excel and later SPSS. IBM SPSS statistics 25 was used for the mathematical analysis, including group comparison, survival analysis and cox regression. A *p*-value less than 0.05 was considered significant.

### Ethical statement

The study was approved by the ethics committee of the Rostock University Medical Center, Rostock, Germany (approval no. A 2020-0188). The CEDATA-GPGE registry was approved by the ethics committee of the University Medicine in Giessen, Germany (approval no. 07/11).

## Results

**Figure 1:**
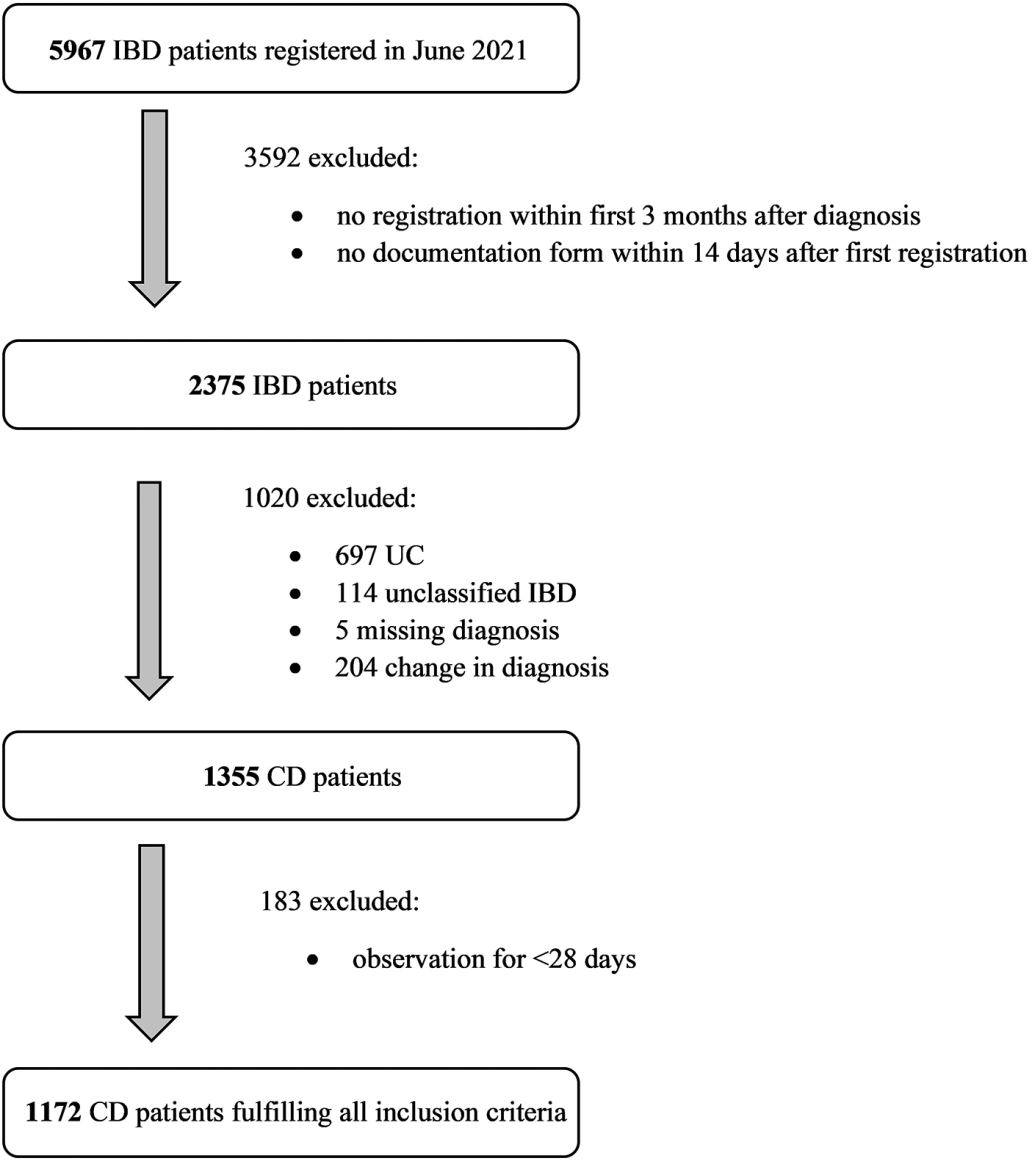
Inclusion of patients in the study. The flow chart demonstrates the inclusion criteria with absolute frequencies. IBD, inflammatory bowel disease; CD, Crohn’s disease; UC, ulcerative colitis.

**Figure 2:**
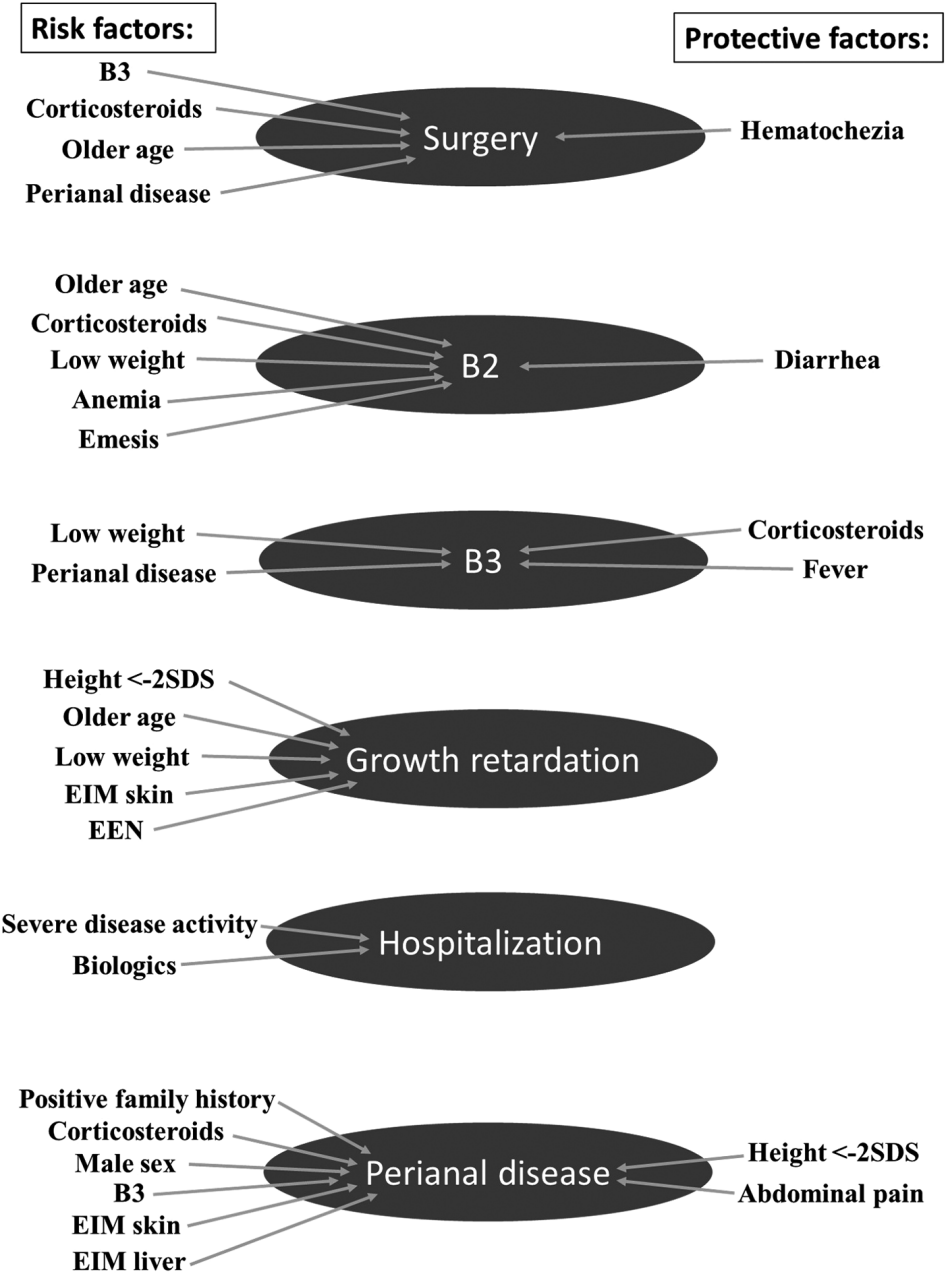
Overview of significant results. This overview presents all identified risk factors and protective factors for possible complications in pediatric Crohn's disease. EEN, Exclusive enteral nutrition therapy; EIM, extraintestinal manifestation; SDS, standard deviation score.

Considering all inclusion criteria 1,172 patients of originally 5,967 patients could be included in the study (Figure 1). The study analyzed 12,658 follow-up-visits with an average patient observation time of 1,119 days (approximately 3 years). The date of diagnosis ranged from November 2003 to April 2021. Only two of 1,172 patients of the cohort suffered from bone disease during follow-up. Therefore, this complication was not further considered. The incidence of bone disease is probably underestimated due to missing systematic observation using for example bone density measurements. Table 1 shows the patient characteristics at the time of diagnosis. Table 2 shows an overview of complications and their respective identified predictors with *P* < 0.05, including absolute frequencies, *P*-value and Hazard ratios. Figure 2 presents an overview of all identified risk factors and protective factors.

**Table 1:** Overview of the characteristics of the patient population at the time of diagnosis.

Characteristics	*N* (%)
Sex	
Male	688 (58.7)
Female	484 (41.3)
Age	
0–2 years	10 (0.9)
3–5 years	38 (3.2)
6–12 years	545 (46.5)
13–17 years	579 (49.4)
Disease activity	
Remission	101 (8.6)
Low	365 (31.1)
Moderate	496 (42.3)
Severe	125 (10.7)
Data not available	85 (7.3)
Disease location involving upper gastrointestinal tract (L4)	
Present	600 (51.2)
Not present	526 (44.9)
Data not available	46 (3.9)
Growth parameters	
Height < −2 SDS	79 (6.7)
Height ≥ −2 SDS	1,078 (92.0)
Data not available	15 (1.3)
Body weight < −2 SDS	201 (17.1)
Body weight ≥ −2 SDS	961 (82.0)
Data not available	10 (0.9)
Body mass index < −2 SDS	231 (19.7)
Body mass index ≥ −2 SDS	925 (78.9)
Data not available	16 (1.4)
Growth retardation	121 (10.3)
No growth retardation	1,051 (89.7)
Disease behaviour	
B1 (inflammatory, non-stricturing, non-penetrating)	950 (81.1)
B2 (stricturing)	22 (1.9)
B3 (penetrating)	41 (3.5)
Data not available	159 (13.5)
Perianal disease	
Stage 1	924 (78.8)
Stage 2	130 (11.1)
Stage 3	13 (1.1)
Stage 4	51 (4.4)
Stage 5	11 (0.9)
Data not available	43 (3.7)
Extraintestinal manifestations	
Not present	944 (80.5)
Present	228 (19.5)
eye	11 (0.9)
joint	65 (5.6)
pancreas	1 (0.1)
skin	60 (5.1)
liver	17 (1.5)
Initial therapy for induction of remission (often combination of two or three)	
Corticosteroids	429 (36.6)
Biologics	47 (4.0)
Exclusive enteral nutrition	206 (17.6)
Azathioprine	337 (28.8)
Methotrexate	18 (1.5)
6-Mercaptopurine	1 (0.1)
Mesalamine	502 (42.8)
Family history of IBD	
Positive	525 (44.8)
Negative	603 (51.4)
Data not available	44 (3.8)
Symptoms	
Not present	2 (0.2)
Present	1,150 (98.1)
abdominal pain	910 (77.6)
activity limitations	440 (37.5)
anemia	283 (24.1)
hematochezia	400 (34.1)
diarrhea	783 (66.8)
fever	186 (15.9)
lip and mouth involvement	52 (4.4)
loss of appetite	267 (22.8)
emesis	69 (5.9)
weight loss	700 (59.7)
Data not available	20 (1.7)

**Table 2:** Overview of complications and their respective identified predictors with *P* < 0.05, including absolute frequencies, *P*-value and hazard ratios.

Complication	Predictor	Complication (Comp) and Predictor (Pred) Frequencies (*n*)				Total (*n*)	*P*-value	Hazard Ratio (CI)
		Comp+ Pred+	Comp+ Pred−	Comp− Pred+	Comp− Pred−			
Surgery								
	**Age**							
	0–2 years	0		10		1172		
	3–5 years	3		35			**.** **031**	**.208** [.050–.865]
	6–12 years	72		473			**<.001**	**.490** [.344–.698]
	13–17 years (ref)	81		498				
	**B3 disease behavior**	13	143	28	988	1172	**.** **044**	**2.002** [1.020–3.932]
	**Perianal disease^b^**							
	Stage 1 (ref)	99		825		1129		
	Stage 2	16		114				
	Stage 3	4		9			**.** **041**	**3.040** [1-044–8.847]
	Stage 4	25		26			**<.001**	**4.738** [2.918–7.695]
	Stage 5	3		8			**.** **002**	**6.331** [1.962–20.431]
	**Corticosteroids**	69	87	360	656	1172	**.** **007**	**1.599** [1.134–2.254]
	**Hematochezia**	37	119	363	653	1172	**<.001**	**.497** [.336–.736]
**Stricturing (B2) disease behavior**								
	**Age**							
	0–2 years	0		10		1172		
	3–5 years	1		37			**.** **025**	**.096** [.012–.744]
	6–12 years	20		525			**<.001**	**.181** [.093–.349]
	13–17 years (ref)	38		541				
	**Corticosteroids**	31	28	398	715	1172	**.** **004**	**2.185** [1.278–3.735]
	**Low weight-for-age**	20	38	181	923	1162	**.** **003**	**2.289** [1.324–3.958]
	**Anemia**	24	35	259	854	1172	**.** **009**	**2.022** [1.195–3.421]
	**Diarrhea**	32	27	751	362	1172	**.** **004**	**.462** [.273–.781]
	**Emesis**	5	54	64	1049	1172	**.** **043**	**2.666** [1.032–6.888]
**Penetrating (B3) disease behavior**								
	**Low weight-for-age**	20	63	181	898	1162	**.** **005**	**2.314** [1.289–4.155]
	**Growth retardation**	4^a^	80	117	971	1172	**.** **006**	**.224** [.077–.648]
	**Perianal disease^b^**							
	Stage 1 (ref)	39		885		1129		
	Stage 2	14		116			**<.001**	**3.097** [1.644–5.836]
	Stage 3	1		12			** **	
	Stage 4	24		27			**<.001**	**19.806** [11.233–34.923]
	Stage 5	3		8			**.** **001**	**10.777** [2.508–46.316]
	**Corticosteroids**	23	61	406	682	1172	**.** **005**	**.461** [.270–.790]
	**Fever**	11	73	175	913	1172	**.** **035**	**.436** [.202–.943]
**Growth retardation**								
	**Low weight-for-age**	88	65	113	896	1162	**<.001**	**2.914** [1.964–4.322]
	**Height < −2SDS**	73	80	6	998	1157	**<.001**	**6.915** [4.703–10.168]
	Age							
	0–2 years	5		5		1172		
	3–5 years	7		31				
	6–12 years	77		468			**<.001**	**.468** [.319–.687]
	13–17 years (ref)	64		515			** **	
	**EEN**	27	126	179	840	1172	**.** **030**	**1.622** [1.049–2.509]
	**EIM skin**	9	144	51	966	1170	**.** **002**	**3.102** [1.528–6.297]
**Hospitalization**								
	**Disease activity**							
	Remission (ref)	30		71		1087		
	Low	107		258				
	Moderate	165		331				
	Severe	69		56			**<.001**	**2.406** [1.560–3.711]
	**Biologics**	24	380	23	745	1172	**.** **017**	**1.805** [1.111–2.930]
**Perianal disease**								
	**Sex**							
	Male	186		502		1172	<.001	**1.670** [1.270–2.195]
	Female	79		405				
	**Corticosteroids**	111	154	318	589	1172	**.** **012**	**1.410** [1.080–1.840]
	**Height < −2SDS**	11	250	68	828	1157	**.** **042**	**.532** [.290–.977]
	**Penetrating (B3) disease behavior**	28	237	13	894	1172	**<.001**	**7.523** [4.879–11.599]
	**Positive family history**	142	113	383	490	1128	**.** **006**	**1.434** [1.107–1.857]
	**Abdominal pain**	184	81	726	181	1172	**.** **002**	**.653** [.499–.855]
	**EIM liver**	7	258	10	897	1172	**.** **007**	**2.836** [1.327–6.064]
	**EIM skin**	20	245	40	865	1170	**.** **023**	**1.745** [1.078–2.823]

Ref, reference when analyzing variables with multiple categories; CI, confidence interval; EEN, exclusive enteral nutrition; EIM, extraintestinal manifestation; PSC, primary sclerosing cholangitis.

^a^ Not considered because of less than five patients who were positive for predictor and complication.

^b^ Perianal disease: stage 1, inconspicuous anal finding; stage 2, rhagades and fissures; stage 3, inactive fistula; stage 4, secreting fistula, abscess or inflammatory induration; stage 5, multiple inflammable anal folds.

### Predictors of surgery

**Figure 3:**
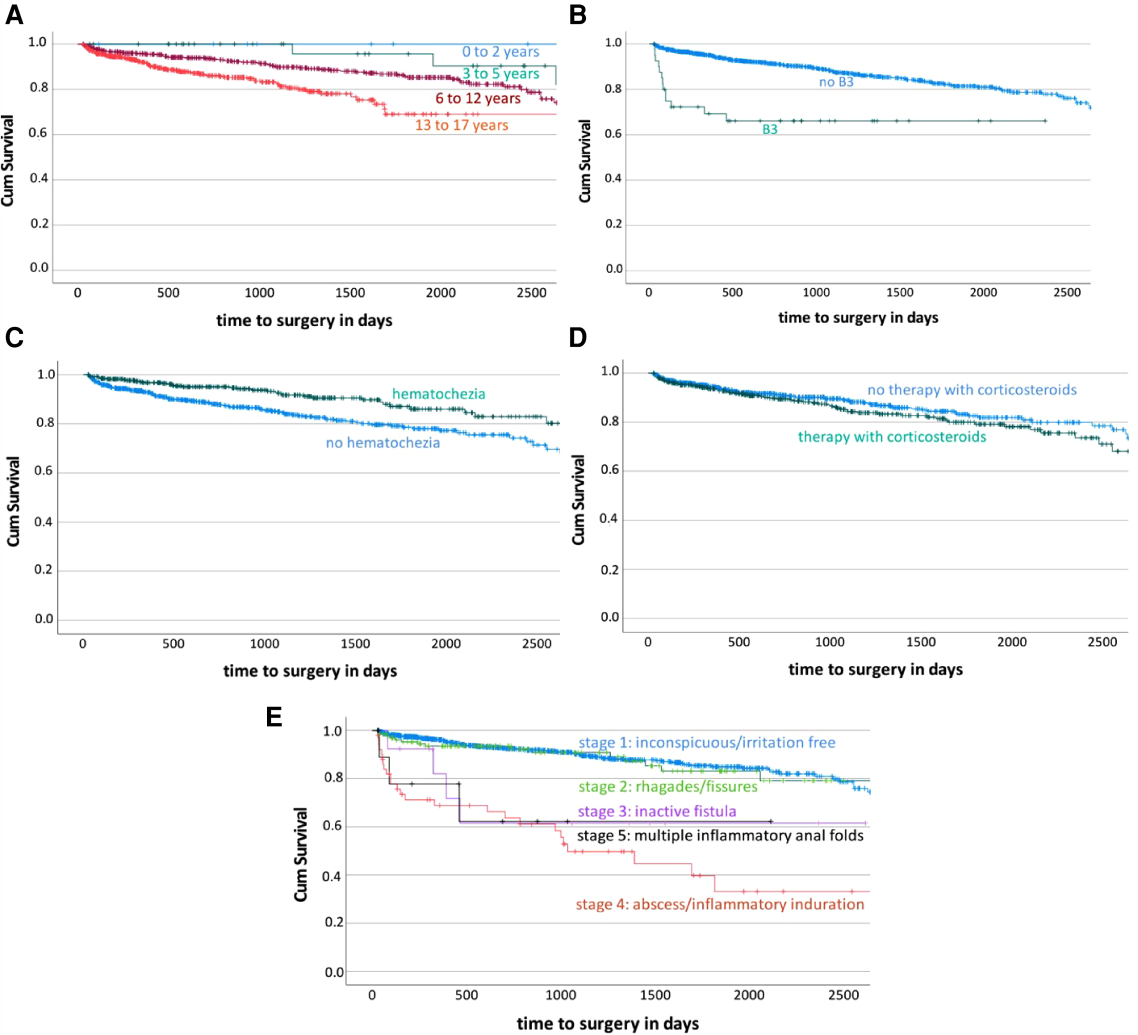
Kaplan Meier survival curves according to the complication “surgery”. The Kaplan Meier survival curves demonstrate the development of the ratio of patients with the need of surgery during disease course over time stratified by the predictors age, penetrating (B3) disease, hematochezia, corticosteroid therapy and perianal disease. 3A predictor age; 3B predictor B3 disease; 3C predictor hematochezia; 3D predictor corticosteroids; 3E predictor perianal disease. Cum survival = ratio of patients without the complication surgery during disease course.

156 (13.3%) patients out of 1,172 had to undergo surgery. Five predictors were identified to be related with an increased risk for surgery (Table 1 and Figures 3A–E). The Kaplan-Meier survival curve illustrates that the higher the patients age at diagnosis, the more likely was the necessity of surgery later on (Figure 3A). Regarding the cox regression, age group 2 (3–5 years; 7.9% needed surgery) showed a significant difference to group 4 (13–17 years; 14.0%; *P* = .031). Furthermore, there was a significant difference between age group 3 (6 to 12; 13.2%) and age group 4 (*P* < .001). Only 10 children belonged to age group 1 (0–2 years) and none of them suffered from this complication. Patients with a penetrating disease behavior (B3) at the time of diagnosis had a higher likelihood of surgery (31.7%; *P* = .044) compared to patients who had no penetrating disease behavior (12.6%). The Kaplan-Meier survival curve shows that the more severe the anal findings at the time of diagnosis, the more likely is the chance for surgery (Figure 3E). These results are confirmed by the multivariate cox regression. Thereby, an inactive fistula (30.8%; *P* = .041), a secreting fistula, abscess, or inflammatory induration (49%; *P* < .001) as well as multiple inflammable anal folds (27.3%; *P* = .002) could be identified as predictors for surgery compared to an inconspicuous anal finding at diagnosis (10.7%). Patients treated with corticosteroids at the beginning of the disease course had to undergo surgery more often (16.1%; *P* = .007) than patients without corticosteroid treatment (11.7%). Patients with hematochezia at the time of diagnosis were less likely to need surgery later on (9.3% vs. 15.4%; *P* < .001).

### Predictors of stricturing (B2) disease behavior

**Figure 4:**
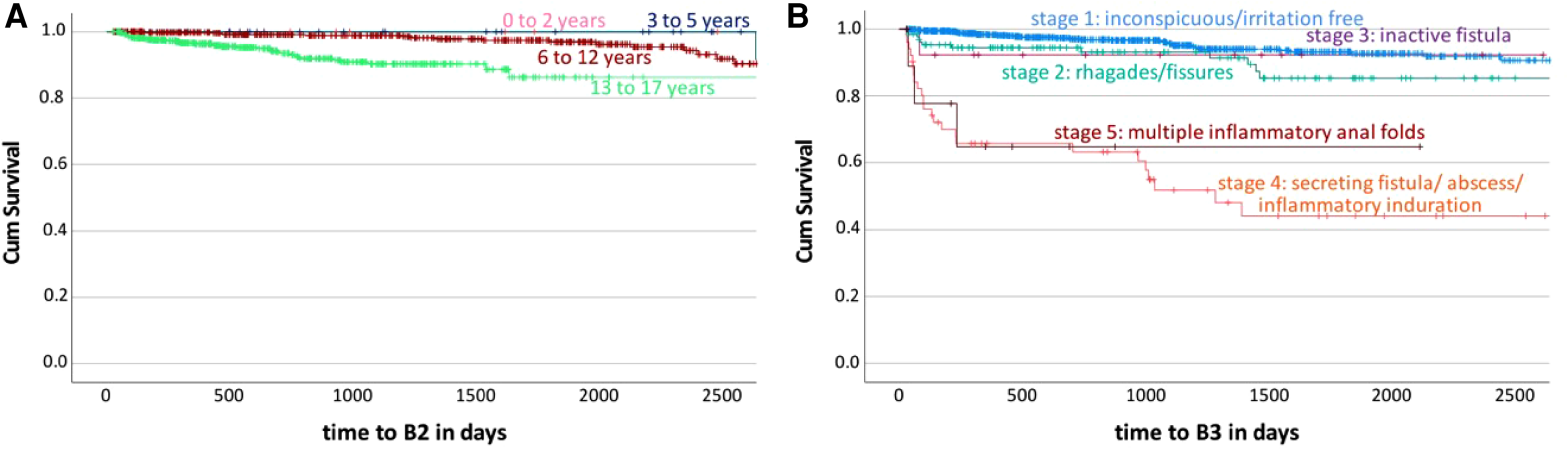
Kaplan Meier survival curves according to the complications “stricturing disease behavior (B2)” and “penetrating disease behavior (B3)”. The Kaplan Meier survival curves demonstrate the development of the ratio of patients developing a stricturing (B2) or penetrating (B3) behavior during disease course over time stratified by the predictors age and perianal disease. 4A predictor age according to complication “B2”; 4B predictor perianal disease according to complication “B3”. Cum survival = ratio of patients without the complication “B2” or “B3” during disease course.

59 (5.0%) out of 1,172 patients developed stricturing disease. Five predictors and one protective factor were identified for a B2 behavior (Table 1 and Figure 4A). Children and young adolescents with low weight-for-age at the time of diagnosis had a higher chance of developing a B2 behavior during the disease course (10.0% vs. 4.0%; *P* = .003). The comparison of the age groups of 3–5 years and 6 to 12 years with 13–17 years showed a significant higher risk in the older group (2.6% and 3.7% vs. 6.6%; *P* = .025, *P* < .001, Figure 4A). Patients treated with corticosteroids at the beginning of their disease were more likely to have a B2 disease behavior (7.2% vs. 3.8%; *P* = .004). An anemia at disease onset was identified as a predictor of B2 behavior (8.4% vs. 3.9%; *P* = .009). Patients who initially suffered from diarrhea were less likely to have a B2 behavior during the disease course (4.1% vs. 6.9%; *P* = .004). Children and young adolescents who suffered from emesis at disease onset had a higher chance to develop a B2 behavior (7.2% vs. 4.9%; *P* = .043).

### Predictors of penetrating (B3) disease behavior

84 (7.2%) out of 1,172 patients developed a penetrating disease. Two risk factors and two protective factors were identified with an increased risk for a B3 behavior (Table 1 and Figure 4B). Patients with low weight-for-age at the time of diagnosis had a higher chance to develop a B3 behavior (10.0% vs. 6.6%; *P* = .005). The multivariate cox regression indicated growth retardation at disease onset as a protective factor, but due to the small patient number of only four children affected by growth retardation and B3 behavior, no statistical analysis was performed. The Kaplan-Meier survival curve shows that the more severe the perianal disease at the beginning of disease, the more likely is the chance of a B3 behavior later on (Figure 4B). These results were confirmed by the multivariate cox regression where rhagades and fissures (10.8%; *P* < .001), a secreting fistula, abscess or inflammatory induration (47.1%; *P* < .001) as well as multiple inflammable anal folds (27.3%; *P* = .001) were identified as predictors. Treatment with corticosteroids at disease onset was identified as a protective factor for the development of a B3 behavior (5.4% vs. 8.2%; *P* = .005). Children and young adolescents who suffered from fever at the disease onset were less likely to develop a B3 behavior during disease course (5.9% vs. 7.4%; *P* = .035).

### Predictors of growth retardation

153 (13.0%) out of 1,172 patients suffered from growth retardation during their disease course. Five predictors were identified with an increased risk for growth retardation (Table 1). Patients with low weight-for-age at disease onset had a higher chance of developing a growth retardation during the observation period (43.8% vs. 6.8%; *P* < .001). Children and young adolescents whose height was at least two standard deviations lower than the average at diagnosis had a much higher chance to also suffer from growth retardation later (92.4% vs. 7.4%; *P* < .001). Younger age groups at the time of diagnosis had a lower risk for developing growth retardation. In the multivariate cox regression, age group 3 (6–12 years at diagnosis) showed a significantly lower risk to develop growth retardation compared to group 4 (13–17 years at diagnosis; *P* < .001). Patients treated with EEN right at the beginning of disease had a higher risk for growth retardation during disease course (*P* = .030) in our analysis. EIM of the skin was identified as a predictor of growth retardation (15.0% vs. 13.0%; *P* = .002). 92.2% of patients who suffered from growth retardation during disease course belonged to age group 6–12 years or 13–17 years at diagnosis. Patients treated with EEN at disease onset had an average height standard deviation score (SDS) at diagnosis of −0.46, whereas patients without EEN therapy had an average height SDS of −0.35 at diagnosis.

### Predictors of hospitalization

404 (34.5%) out of 1,172 patients had at least one hospital stay. Two predictors were identified with an increased risk for a stay in hospital (Table 1). Children and young adolescents with severe disease activity at onset had a higher chance to be hospitalized compared to children who had the lowest level of disease activity (55.2% vs. 29.7%; *P* = .001). Treatment with biologics at the beginning of disease was identified as a predictor of hospitalization (51.1% vs. 33.8%; *P* = .017). In our study 47 patients (4%) were initially treated with biologics (mainly infliximab or adalimumab, *N* = 38).

### Predictors of perianal disease

265 (21.8%) out of 1,172 patients suffered from perianal disease during the disease course. Six risk factors and two protective factors were identified for perianal disease (Table 1). Male patients had a higher chance to develop perianal disease compared to female patients (27.0% vs. 16.3%; *P* < .001). A penetrating disease behavior was identified as a risk factor for perianal disease (68.3% vs. 21.0%; *P* < .001). Children and young adolescents who were treated with corticosteroids at the beginning of their disease had a higher chance to develop perianal disease (25.9% vs. 20.7%; *P* = .012). Patients who had abdominal pain at the time of diagnosis were less likely to have perianal disease later on (20.2% vs. 30.9%; *P* = .002). A positive family history was identified as a predictor of perianal disease (27.0% vs. 18.7%; *P* = .006). Patients who suffered from EIM of the skin (33.3% vs. 22.1%; *P* = .023) or liver (41.2% vs. 22.3%; *P* = .007) had a higher chance to develop perianal disease. Growth impairment at diagnosis could be identified as a protective factor for the development of perianal disease (13.9% vs. 23.2%; *P* = .042).

### Additional calculations regarding disease activity at diagnosis

13.6% of patients with severe disease activity at disease onset were initially treated with biologics, whereas only 1.6% and 2.8% of patients with low and moderate disease activity received biologics. The average time until the first follow-up meeting was shorter the higher the level of disease activity (low activity 138 days, moderate activity 118 days, severe activity 66 days).

## Discussion

The present study investigated predictors for specified complications of pediatric CD using data from the CEDATA-GPGE registry. A strength of the study is the high number of 1,172 patients and 12,658 follow-up visits. Patients were observed for 43,716.6 months (approximately 3,643 years). Another strength of the study is that many parameters of different fields (growth retardation, disease location and activity, initial therapy, symptoms, EIMs, patient characteristics) were investigated in one single study. Therefore, it was possible to compare the validity of the predictors and find potential interactions between the parameters. Beside the wide range of parameters that the CEDATA-GPGE registry provides, a further benefit is that patients from all over Germany and Austria are included. Therefore, the registry represents a reliable cross-section of the patient population and treatment in those countries. The registry also shows limitations, for example that patients are only registered until their 18th birthday. Consequently, there was no opportunity to analyze complications that occurred later during the disease course and children who were diagnosed with CD at an older age only had a short observation time.

### Surgery

Two North American studies already identified higher age at disease onset as a predictor of the necessity of surgery during the disease course (10, 11). Our study confirmed this correlation in patients from Germany and Austria. It is known that patients younger than 5 years at disease onset present more often with isolated colonic disease which may be a reason for the decreased risk for surgery (11, 12). Nevertheless, our study also identified a significantly lower risk for surgery for the age group 6–12 years compared to the age group 13–17 years indicating that the older the patient at disease onset, the higher is the risk for surgery. In addition to age, our data reveal that penetrating disease behavior (B3) might be a predictor of surgery, which corresponds to the previously described findings (10, 11, 13, 14). B2 was found to show a trend towards increased risk of surgery, as previously described in the literature (11, 13, 14).

Our study identified perianal disease at diagnosis as a risk factor of surgery, which has also been reported in a very recent study in a small group of 57 patients (14). This is likely because specific types of severe perianal disease can be an indication for surgery (15, 16). According to the results of our study, treatment with corticosteroids is a predictor of surgery. The therapy with corticosteroids has previously been identified as a risk factor of perianal disease in pediatric patients (17) which seems to be a predictor of surgery in our study. In contrast to corticosteroid therapy, nutritional therapy was not a risk factor for surgery in our study. This supports the hypothesis of corticosteroids being less effective considering the mucosal healing and clinical remission compared to EEN (18). Hematochezia was associated with decreased risk for surgery. We speculate that the presentation of severe symptoms at the point of diagnosis might lead to a more intensified initial therapy as well as more regular follow-up appointments, which both might prevent complications due to an early detection and intervention of persistent active disease.

### Stricturing disease behavior (B2)

Stricturing (B2) and penetrating (B3) disease are common types of disease behavior in pediatric CD (19). Our study found higher age at disease onset as a predictor of B2 disease, which has also been reported in a previous study (20). Nevertheless, there are also studies that found no association between age at diagnosis and stricturing disease (21, 22). Ricciuto et al. stated that a meta-analysis of the studies was not possible due to different methods (univariate and multivariate cox regression) and dissimilar age group definitions (6). The largest and according to Ricciuto et al. the only high-quality study found no association between older age and stricturing disease, but instead for penetrating disease (B3) (6, 22). Our results indicate that corticosteroids might be a risk factor for B2 behavior, which has been described before (17). As discussed for the outcome of surgery, corticosteroids are less effective in reaching mucosal healing compared to EEN as induction therapy (18). Vasseur et al. claimed that children and young adolescents with CD who suffer from stricturing disease often show a poor nutritional status (23). This observation is supported by our results demonstrating that low weight-for-age at diagnosis is a risk factor for B2 behavior. In addition to corticosteroid treatment and a low weight-for-age, our study identified anemia as a potential risk factor of B2 behavior, which has not been reported yet. Nevertheless, previous studies associated anemia with higher disease activity as well as lower self-assessed well-being supporting an impact of anemia on the development of a B2 behavior (24). Our study identified emesis as a risk factor and diarrhea as a protective factor of B2 behavior, which has not been reported yet. It is possible that some patients with emesis who do not suffer from diarrhea at diagnosis might already have an undiscovered stenosis or a developing stenosis that will be diagnosed later during the disease course.

### Penetrating disease behavior (B3)

Vasseur et al. related a poor nutritional status at diagnosis with a poor prognosis (23), which can be confirmed in our study, e.g., considering B2 and B3 behavior. Perianal disease at diagnosis is already known as a risk factor for the development of fistulas during disease course (25, 26), which was also confirmed in our study. Initial therapy with corticosteroids was identified as a protective factor for B3 behavior during disease course and as a risk factor for perianal disease and B2 behavior. The results of our study indicate that patients with fever at the time of diagnosis are less likely to develop a B3 behavior during disease course. This has not been reported previously. One explanation may be that patients with severe symptoms at disease onset receive a more potent initial therapy and a closer follow-up. We could confirm that patients with a severe disease activity at diagnosis were more often treated with biologics compared to patients with low and moderate disease activity. The average time until the first follow-up meeting was shorter in patients with a high level of disease activity.

### Growth retardation

It has been reported that children and adolescents with growth failure at diagnosis have a higher risk for growth retardation at the end of observation (27) which was confirmed by our analysis. Furthermore, our results indicate that higher age of children at diagnosis was associated with higher risk for growth retardation, confirming previously reported results (28). Nevertheless, there are also studies suggesting the opposite (29, 30). According to Ricciuto et al. a reason for the contradictory study results are the differing definitions of growth retardation (height <2 standard deviations vs. Z-Score <1.64) (6). Another possible explanation is that patients are more vulnerable for growth retardation during pre-pubertal and pubertal stages due to the fast growth spurt during this period. Our study strengthens the idea of higher age as a risk factor for growth retardation. Together with our finding of an increased risk of surgery, we conclude that diagnosis of CD at higher age might correlate with a higher risk of complications during follow-up. Our study identified low-weight-for-age as risk factor for growth impairment. Due to the fact that we also found low-weight-for-age at diagnosis as a predictor for a B2 and B3 behavior, we can confirm the statement that it might be a strong indicator for a poor prognosis (23) and should always be included in risk stratifications. We also identified EEN as a risk factor for growth impairment. A possible explanation might be that patients at the threshold to low-weight-for-age and growth retardation at the point of diagnosis are more likely to get EEN. We could confirm that patients treated with EEN at disease onset had a lower height at diagnosis compared to patients who were not treated with EEN (SDS −0.46 vs. −0.35). We suppose that despite of an EEN therapy, those patients are more likely to cross this threshold compared to children who are normal weight. Furthermore, it might be possible that patients who are treated with EEN are sometimes undersupplied with macronutrients due to underestimation of energy requirements or deficient compliance. It is important that there is a sufficient nutrient supply provided. One study from 2010 identified EIM as a predictor of poor prognosis (23). Nevertheless, future studies are needed to further classify these findings. We analyzed single EIM in detail and found a significant correlation between skin manifestations at the time of diagnosis and growth impairment as a later complication. There are different types of skin manifestations that can occur, for example an erythema nodosum, a pyoderma gangrenosum or purpura (31). Especially purpura, angular cheilitis as well as hair and nail abnormalities can be caused by malnutrition (31). Therefore, an explanation for the correlation between EIM of the skin at diagnosis and growth impairment as a complication might be that the skin diseases are an early sign of malnutrition and hence growth impairment. We could not confirm EIMs in general as a risk factor for growth retardation.

### Hospitalization

The initial therapy with biologics is a risk factor for hospitalization. Only children and adolescents with a severe phenotype at the time of diagnosis were initially treated with biologics (32). This severe phenotype is defined by predictors of poor outcome (POPO criteria) and includes deep colonic ulcerations, persistent disease, extensive disease, severe growth retardation, stricturing and penetrating disease and/or perianal disease (27, 19).

We also identified that patients with a severe disease activity at diagnosis have a higher risk for hospitalization. Hence, it might be possible that not the therapy with biologics itself is the reason for hospitalization but the initially more severe form of the disease. However, in a multivariate cox regression only independent variables turn out to be significant. Hence, the disease activity at diagnosis and the necessity of an initial therapy with biologics are both identified as independent risk factors for hospitalization in our study. The observed increased risk for hospitalization after the initial treatment with biologics may also be a consequence of adverse side effects. A known side effect is the acute infusion reaction that is associated with autoantibodies developed to Infliximab, which effects about 8% of patients, especially girls (33, 34). Nevertheless, the risk for delayed infusion reactions, other autoimmune phenomena and serious infections is very low in children with IBD (34).

### Perianal disease

Male sex and initial therapy with corticosteroids have already been identified as risk factors for perianal disease in previous studies (17, 35) and were confirmed by our analysis. Furthermore, we identified growth retardation and abdominal pain at disease onset as protective factors. A possible explanation might be that patients with those symptoms at disease onset are treated with a more potent therapy and closer follow-up. Our results reveal that penetrating disease at disease onset is a risk factor for perianal disease later on. Since perianal disease is also known as a risk factor for a penetrating disease behavior, we assume that there is a strong correlation between those two factors. Brückner et al. analyzed 742 patients of the CEDATA-GPGE registry, who were diagnosed with CD from 2004 to 2014. In this study EIM and a positive family history could not be identified as risk factors for perianal disease (17). In contrast, our study, including 1,172 patients from 2003 to 2021, found a significant correlation between a positive family history and perianal disease, which might be attributed to the larger patient population and the slightly differing definition of perianal disease. In the study of Brückner et al. only the appearance of perianal abscesses and fistulas were defined as perianal disease, whereas in our study also anal eczemas, perianal lesions and the assessment of perianal disease higher than stage 2 were included (17). In contrast to Brückner et al. (incidence of perianal disease 9.8% initially) we found an incidence of 22.6% during disease course (17). This findings are in accordance with previous studies that identified incidences of 21% (35) and 27% (13). In addition to Brückner et al., we did also analyze specific EIM separately. Here, we identified skin as well as liver manifestations as risk factors for perianal disease. A possible explanation for the correlation between liver manifestations and perianal disease might be that both seem to be associated with a higher level of inflammation.

## Conclusion

In this study, we confirmed the factors male sex, higher age, penetrating disease (B3), perianal disease, corticosteroids, low weight-for-age and growth retardation at diagnosis as predictors for selected complications. In addition, we did also identify hematochezia and fever as new protective factors and skin and liver manifestations as well as higher disease activity, initial biologics, emesis, and the absence of diarrhea as new risk factors for complications. However, more studies with different cohorts are needed to further strengthen and clarify the most important and most helpful predictors. Finding reliable predictors of complications is only the first step. In the second step it is necessary to identify specific initial therapies that could alleviate the risk carried by specific predictors. Prospective studies with high numbers of patients from different countries, are needed to further classify specific predictors for complications in pediatric CD (36).

## Data Availability

The raw data supporting the conclusions of this article will be made available by the authors, upon reasonable request.

## Collaborators

CEDATA-GPGE study group: Stephan Buderus, Bonn, Germany; Philip Bufler, Berlin, Germany; Martin Claßen, Bremen, Germany; Jan Däbritz, Greifswald, Germany; Söhnke Dammann, Stuttgart, Germany; Jan de Laffolie, Giessen, Germany; Almuthe Christina Hauer, Graz, Austria; Klaus-Michael Keller, Wiesbaden, Germany; Andreas Krahl Darmstadt, Germany; Martin Laaß, Dresden, Germany; Thomas Lang, Regensburg, Germany; Carsten Posovszky, Zürich, Switzerland; Burkhard Rodeck, Osnabrück, Germany; Stefan Trenkel, Potsdam, Germany.
