# Supplementary material for: Predicting complications in pediatric Crohn's disease patients followed in CEDATA-GPGE registry
Source: Front Pediatr. 2023 Feb 15;11:1043067. doi: 10.3389/fped.2023.1043067 (PMC9975712; doi:10.3389/fped.2023.1043067)
Supplement: Supplementary file 1 [file Table1.docx]

Supplementary Material

# Supplementary Table 1. Definitions of predictors for complications in pediatric CD patients.

| **Predictor** | **Definition** |
| --- | --- |
| Growth retardation | Height <-2 standard deviations than the average  Assessment by pediatric gastroenterologist |
| Body Mass Index (BMI) | <-2 standard deviations than the average |
| Low weight-for-age | <-2 standard deviations than the average |
| Disease location | Paris Classification (1)   - L1: distal ileum and caecum - L2: colon - L3: terminal ileum and colon - L4: upper gastrointestinal tract   L4 at diagnosis: yes/no |
| Age | - Group 1: 0-2 years - Group 2: 3-5 years - Group 3: 6-12 years - Group 4: 13-17 years |
| Disease behavior | Paris Classification (1)   - B2: stricturing disease - B3: penetrating disease |
| Disease activity | Assessment by pediatric gastroenterologist   - 1: remission - 2: low activity - 3: moderate activity - 4: severe activity |
| Perianal disease | Assessment by pediatric gastroenterologist   - Stage 1: inconspicuous/irritation free - Stage 2: rhagades/fissures - Stage 3: inactive fistula - Stage 4: abscess/inflammatory induration - Stage 5: multiple inflammatory anal folds |
| Family history | - Positive: at least one biological relative also suffered from Crohn’s disease |

# Supplementary References

1. Levine A, Griffiths A, Markowitz J, Wilson DC, Turner D, Russell RK et al. Pediatric modification of the Montreal classification for inflammatory bowel disease: the Paris classification. Inflamm Bowel Dis 2011; 17(6):1314–21.
